# Ochratoxin A and AFM1 in Cheese and Cheese Substitutes: LC-MS/MS Method Validation, Natural Occurrence, and Risk Assessment

**DOI:** 10.3390/toxins16120547

**Published:** 2024-12-18

**Authors:** María Agustina Pavicich, Stefano Compagnoni, Celine Meerpoel, Katleen Raes, Sarah De Saeger

**Affiliations:** 1 Centre of Excellence in Mycotoxicology and Public Health, Department of Bioanalysis, Faculty of Pharmaceutical Sciences, Ghent University, B-9000 Ghent, Belgiumsarah.desaeger@ugent.be (S.D.S.); 2 Research Unit VEG-i-TEC, Department of Food Technology, Safety and Health, Faculty of Bioscience Engineering, Ghent University Campus Kortrijk, B-8500 Kortrijk, Belgium; katleen.raes@ugent.be; 3 Department of Biotechnology and Food Technology, Faculty of Science, University of Johannesburg, Doornfontein Campus, Gauteng 2028, South Africa

**Keywords:** cheese, mycotoxins, food safety, Parmigiano Reggiano

## Abstract

Cheese is vulnerable to contamination with mycotoxins, particularly ochratoxin A (OTA) and aflatoxin M1 (AFM1). This study aims to develop and validate an analytical method for the detection and quantification of OTA and AFM1 in cheese and to assess their prevalence and associated risks. A liquid chromatography–tandem mass spectrometry (LC-MS/MS) method was validated for detecting these mycotoxins in 41 cheese samples, including firm-ripened, spreadable, and plant-based alternatives. The results showed that OTA was detected exclusively in grated Parmigiano Reggiano cheese, while AFM1 was found in both Parmigiano Reggiano and Pecorino cheeses. This study goes beyond analytical method development by providing a preliminary exposure assessment and risk characterization for OTA and AFM1 in cheese, bridging the gap between analytical chemistry and public health implications. This study identified potential health risks associated with OTA, particularly for children and adolescents categorized as high consumers of Parmigiano Reggiano cheese. The findings underscore the need for monitoring of OTA and AFM1 in cheese and further research to establish regulatory limits for these contaminants.

## 1. Introduction

Cheese, a staple food in many cultures worldwide, is not only esteemed for its diverse flavors and textures but also recognized for its nutritional value. Nonetheless, chemical contaminants such as polycyclic aromatic hydrocarbons, dioxins, metals, and antibiotic and pesticide residues were found in different varieties of cheese, posing health risks [1,2]. Moreover, filamentous fungi are capable of growing on all sorts of cheeses with a beneficial purpose (e.g., to ripen, or add flavor) or causing undesired spoilage and a potential health risk for consumers [3,4,5]. When fungi are not added intentionally, unwanted changes in the cheese might occur. Some of the genera that have been reported to cause problems in the cheese industry are *Acremonium, Alternaria, Aspergillus, Aureobasidium, Botrytis, Cladosporium, Epicoccum, Eurotium, Fusarium, Mucor, Rhizopus,* and *Penicillium* [6]. Several species of *Penicillium* have been reported as the main undesired fungi in firm, semi-firm, and semi-soft cheeses [7]. Moreover, some of these fungi are able to produce mycotoxins, secondary metabolites produced by various fungal species that can contaminate food and feed, posing significant risks to human and animal health. The presence of mycotoxins in cheese has raised concerns regarding food safety and public health [5,8,9]. Cheese provides an ideal substrate for mycotoxin contamination and accumulation. During cheese production, factors such as the use of contaminated milk, environmental conditions, and storage practices can facilitate the growth of mycotoxin-producing fungi and the transfer of mycotoxins into the cheese matrix [10,11]. The diverse range of cheese types, including firm-ripened cheeses like Parmesan and Pecorino, and spreadable cheeses like Camembert, provides varying environments that may influence mycotoxin contamination levels and distribution patterns. Even if the fungi are eliminated from the surface of the cheese, mycotoxins can still be present in the final product [12]. This is of particular relevance in grated cheese, where the rind of the cheese is sometimes removed, eliminating the fungi, but mycotoxins can still remain.

On the other hand, cheese substitutes, a growing segment in the market, are increasingly consumed as an alternative to traditional dairy cheese [13]. Unlike dairy cheese, cheese substitutes are made from plant-based ingredients such as nuts, seeds, soy, or root vegetables. These ingredients can still be susceptible to mycotoxin contamination, particularly if the raw materials used are exposed to mycotoxin-producing fungi during cultivation, storage, or processing. This underlines the importance of considering mycotoxin contamination in both dairy and cheese substitute products.

Among these mycotoxins, ochratoxin A (OTA) and aflatoxin M1 (AFM1) have gathered significant attention due to their occurrence in cheese and their potential adverse effects on human health. OTA is associated with carcinogenic and immunotoxic effects, and with Balkan endemic nephropathy, chronic interstitial nephropathy, and other renal diseases, and is classified as group 2B (possible carcinogen to humans) by the International Agency for Research on Cancer [14,15,16]. AFM1 is known for its hepatotoxic and carcinogenic properties, even though aflatoxin B1 is 10 times more potent than AFM1 with respect to liver carcinogenicity [17,18]. OTA is primarily produced by *Aspergillus* and *Penicillium* species, while AFM1 is a metabolite of aflatoxin B1, produced by *Aspergillus flavus* and *Aspergillus parasiticus*. With Commission Regulation 2023/915, the European Union established a maximum limit for AFM1 in milk and insisted on the importance of defining enrichment factors (EFs) for cheese, but, to date, there is no regulation for OTA or AFM1 in dairy cheese [19,20].

Given the potential health risks associated with OTA and AFM1 exposure, there is a critical need for reliable analytical methods to detect and quantify these mycotoxins in cheese and cheese substitutes. The aim of this study was to develop and validate an extraction method followed by a liquid chromatography–tandem mass spectrometry (LC-MS/MS) method for the detection and quantification of OTA and AFM1 in cheese. With the validated method, 40 commercially available samples were analyzed, and, for the first time, an exposure assessment and risk characterization of both mycotoxins were made for the Belgian population.

## 2. Results and Discussion

### 2.1. Method Validation

The performance of the method was demonstrated through the coefficient of determination (R^2^), limit of detection (LOD), the calibration range expressed as the lower limit of quantification (LLOQ)–upper limit of quantification (ULOQ) interval, the signal suppression/enhancement (SSE, %), recovery (%), repeatability (RSD_r_, %), and intermediate precision (RSD_R_, %) (Table 1). Regarding the suitability of the method and its validation, all the parameters satisfied the current guidelines for the quantification of mycotoxins in food samples [19,20]. The signal suppression was −83 for OTA and −66 for AFM1. For OTA, the LOD was 0.1 μg/kg and the LLOQ 0.34 μg/kg, with a linear range up to 20.00 μg/kg. The method achieved low LODs and LOQs for both OTA, enabling reliable quantification at trace levels. The recoveries were 104.9% and 101.8% in the lowest and highest calibration points. For AFM1, the LOD was 0.02 μg/kg, the LLOQ 0.04 μg/kg, and the ULOQ 2 μg/kg; the linear range was from 0.01 to 2.00 μg/kg. The recovery was 104.6% and 101.8% for the LLOQ and ULOQ, respectively. Repeatability (RSD_r_) and intermediate precision (RSD_R_) were below 20% for all the calibration points for both mycotoxins. The validation results demonstrate robust linearity and goodness-of-fit, with R^2^ above the acceptance threshold (>0.990). No interference peaks and carry-over were detected at the retention time ± 2.5% of the analytes with S/N values ≥3.

### 2.2. Occurrence of OTA and AFM1 in Different Varieties of Cheese

With the validated method, 41 commercially available cheese samples corresponding to spreadable cheese, cheese substitutes, and firm-ripened cheese were analyzed. To our knowledge, this is the first study to report the occurrence of OTA and AFM1 in cheese products marketed in Belgium. Table 2 shows the prevalence, range, and mean concentrations by cheese category. In this study, a total of sixteen spreadable cheeses, seven cheese substitutes, and eighteen varieties of firm-ripened cheese were analyzed for the presence of OTA and AFM1. Notably, all spreadable cheeses and cheese substitutes tested were below the LOD for OTA and AFM1. In spreadable cheese, this can be explained by low levels of OTA and AFM1 in the milk used for the production of these cheeses and the low concentration factor (1.71) from milk to cheese [17]. The non-detection of the targeted mycotoxins in cheese substitutes could be attributed to the ingredients used in the production process of these products and the absence of a ripening period. The main ingredient, shown in Supplementary Material S1, in these products is water, followed by corn starch, and, in general, this matrix was associated with low levels of mycotoxins [21,22,23]. Even though the migration of OTA from raw corn to corn starch was reported to be 32.7%, further processing to make cheese substitutes could influence OTA concentration [23]. Nonetheless, corn starch has been frequently reported to be contaminated with fumonisins, and, in particular, hidden forms of these mycotoxins [24,25]. Thus, further studies on the occurrence of mycotoxins in dairy alternatives such as cheese substitutes should focus on hidden fumonisins.

Regarding the firm-ripened cheese, OTA was exclusively detected in Parmigiano Reggiano cheese, while Pecorino, Grana Padano, and Emmental exhibited levels below the LOD for this mycotoxin. It is worth noting that OTA was only detected in grated Parmigiano Reggiano cheese. In the case of AFM1, it was found in Parmigiano Reggiano and Pecorino cheeses. Our findings align with existing literature, where numerous studies have documented the presence of OTA and AFM1 in ripened cheese [16,26,27]. A recent study by Rodríguez-Cañás et al. found levels of OTA in the range of 4.8 to 9.6 µg/kg [11], while, in Italy, levels from 1.14 to 4.7 µg/kg were found in hard grated cheese [28]. Moreover, Altafini et al. found that grated hard cheeses were contaminated with OTA values ranging between 1.3 and 22.4 µg/kg [29]. In a systematic review, the prevalence of AFM1 in cheese was 49.85%, similar to the results found in this study [30]. The presence of OTA in cheese may arise mainly from environmental contamination during cheese production and maturation processes [31]. Given the minimal transfer of OTA from feed to ruminant milk, attributed to the hydrolytic activity of rumen bacteria, the presence of OTA in cheese is likely a result of environmental contamination fostering fungal growth on the cheese surface, and could be due to the inclusion of the rind in the grated cheese [26]. However, a certain percentage of OTA can bypass the rumen, and this percentage can range from 5% to 62%. As a result, detection of the toxin in milk or cheese is usually limited to situations with high OTA concentrations in the animal’s diet [13,14]. Hence, OTA-producing fungi, such as *Penicillium* and *Aspergillus* species, may colonize the cheese surface and/or penetrate the cheese matrix, leading to the accumulation of OTA in cheese [32,33]. Factors such as temperature, humidity, a_w_, and pH levels during cheese ripening can influence the growth and toxin production of OTA-producing fungi, thereby affecting OTA levels in cheese [30,33,34,35]. Notably, a study on French semi-firm Comté cheese demonstrated the potential migration of OTA from the crust to a depth of 1.6 cm following artificial inoculation with OTA-producing strains [9]. Therefore, the results obtained in this study are in accordance with literature and are more likely to be attributed to environmental contamination [26,27]. In our study, OTA was only detected in grated cheese, emphasizing the need for control strategies to prevent the presence of this mycotoxin in firm-ripened grated cheese. Industrial methods for cleaning cheese wheels and low temperatures in the first stages of ripening can serve as an effective approach to minimize OTA, particularly when grated cheese products are intended [34,35,36,37].

On the contrary, the presence of AFM1 in cheese is primarily attributed to the ingestion of aflatoxin B1-contaminated feed by dairy cows. Following ingestion, aflatoxin B1 is metabolized in the liver of dairy cows, resulting in the excretion of AFM1 in milk. Subsequent cheese production processes, such as curdling and ageing, can concentrate AFM1 in cheese since a significant proportion of AFM1 binds to casein, posing a potential health risk to consumers [17,30]. The EF for firm-ripened cheese is from 4 to 5, resulting in a maximum level of 0.2 to 0.25 µg/kg of AFM1 in firm-ripened cheese [17]. Thus, the analyzed samples would be in accordance with the current regulation. Nonetheless, AFM1 was present in 50% of the analyzed samples, and it is known to be a hepatic carcinogen; thus, an exposure assessment is still justified [16].

### 2.3. Exposure Assessment and Risk Characterization of OTA and AFM1 by the Consumption of Firm-Ripened Cheese

The risk characterization was achieved through the Margin of Exposure (MoE) calculated as BMDL_10_/chronic exposure, based on the BMDL_10_ of 14.5 µg/kg bw/day for OTA and 0.4 µg/kg bw/day and a potency factor of 0.1 for AFM1. Since firm-ripened cheese was the only category in our study that was contaminated with OTA and AFM1, a preliminary deterministic exposure assessment and risk characterization was performed for the Belgian population.

Considering all firm-ripened cheese types, the mean OTA concentration was 0.36 µg/kg and 0.65 µg/kg in the LB and UB, respectively, resulting in MoEs that exceeded 10,000 for all age groups among average and high consumers, thus indicating a low risk of exposure to OTA by the consumption of firm-ripened cheese in Belgium. However, when focusing only on contamination values for Parmesan cheese, the mean OTA concentration in LB was 1.10 µg/kg and 1.26 µg/kg in UB. Consequently, the MoE fell below 10,000 for other children and adolescents categorized as high consumers, indicating a risk for these consumers, while remaining above 10,000 for other consumer groups. This indicates that children and adolescents consuming only Parmesan cheese as firm-ripened cheese are at risk of exposure to higher levels of OTA than the BMDL_10_. There are limited studies on the risk of exposure to OTA in cheese. Nonetheless, EFSA identified cheese as one of the main contributors of exposure [14]. In a global systematic review, meta-analysis and probabilistic risk assessment considering OTA levels in multiple cheese varieties, the MoE was above 10,000 for all countries except for Germany and Greece [30]. Since the presence of OTA depends on the variety of cheese, and consumers select and consume the variety of cheese according to the culinary preparation, separate risk assessments for each variety are recommended.

For AFM1, the mean concentration was 0.01 µg/kg and 0.04 µg/kg in the LB and UB, respectively, resulting in MoEs above the 10,000 for all age groups, indicating a low risk of exposure to AFM1 by the consumption of firm-ripened cheese. A study from Iran also revealed a low risk of exposure to AFM1 by the consumption of cheese [27]. In Serbia, a low health risk related to AFM1 in dairy products was found, but cheese was the biggest contributor to exposure [38]. This was not the case for many other countries, in which the MoE in children was below 10,000 for all but one of the studied countries [30]. It is worth noting that the magnitude of a MoE only indicates a level of concern and does not quantify risk. Moreover, the MoE method relies on the use of BMDL_10_ as a threshold for risk assessment. This assumes a linear dose–response relationship below the reference point, which may oversimplify the actual risk posed by low-dose exposure to mycotoxins, especially given their possible cumulative or synergistic effects. The MoE approach evaluates each mycotoxin independently and does not account for the potential combined or additive effects of OTA and AFM1 nor their interactions with other mycotoxins or chemical contaminants that may be present in the diet. The total risk of exposure to OTA and AFM1 should be evaluated by incorporating other food products and the combined toxicity of mycotoxins.

## 3. Conclusions

This study provides a reliable method to detect and quantify OTA and AFM1 in cheese and valuable insights into their occurrence in Belgium. Understanding the prevalence of these mycotoxins in cheese and the risk associated with its consumption is essential for food producers, regulatory agencies, and risk managers to implement effective measures to minimize exposure and safeguard public health. To our knowledge, this is the first report of exposure to OTA and AFM1 by the consumption of firm-ripened cheese in Belgium, and more research is needed to further evaluate the exposure and to set regulatory limits for these mycotoxins in cheese.

## 4. Materials and Methods

### 4.1. Standards and Reagents

OTA (1 mg, solid standard) and AFM1 (0.502 µg/mL in acetonitrile (MeCN)) were purchased from Fermentek (Fermentek Ltd., Jerusalem, Israel). A liquid standard of ^13^C-labelled OTA (^13^C_20_ OTA) in MeCN was purchased from Biopure-Romer Labs (Tulln, Austria), with a concentration of 10.06 µg/mL. Stock solutions of each mycotoxin were stored in a freezer at −20 °C.

### 4.2. Samples

A total of 41 cheese samples were collected by random sampling between August and September 2023 from various supermarkets in Ghent, Belgium. The samples included a diverse range of cheese types including firm-ripened cheese (*n* = 18), cheese substitutes (*n* = 7), and spreadable cheese (*n* = 16). Specifically, the firm-ripened cheese category included varieties such as Pecorino Romano, Emmental, Parmesan Romano, and grana Padano, while vegan cheese represented plant-based alternatives, and spreadable cheese included melted, fresh, Camembert, and Gruyere varieties. Each sample was systematically numbered and accompanied by detailed information regarding batch number, shop of purchase, brand, expiration date, and ingredients. The cheese samples underwent initial processing by grinding the whole piece, when applicable, or grinding to obtain a similar particle size, and/or a thorough homogenization of the whole package before the subsample was taken. All samples were stored at −20 °C prior to analysis.

### 4.3. Extraction

Sample preparation was based on the first step (extraction/partition process) of the QuEChERS procedure. Each sample was thoroughly homogenized, and a representative portion of 2 g of cheese sample and 8 mL of water were placed into a 50 mL screw cap test tube with a conical bottom, which was shaken by vortex for 10 s. Subsequently, 8 mL of 1% acetic acid in MeCN was added to the tube and shaken by vortex for 2 min. A mixture of salts (4 g MgSO_4_ and 1 g NaCl) was added, and the tube was vigorously shaken by hand for 1 min and by vortex for 2 min. Then, a centrifugation step was performed at 4500 rpm for 5 min at 4 °C, and 5 mL of the supernatant was transferred into a tube, and 2 mL of hexane was added. The tube was vortexed for 2 min and centrifugated for 5 min at 4500 rpm. The upper layer was discarded, and the MeCN layer was evaporated to near dryness under a gentle stream of N_2_ at 40 °C. The dried extract was reconstituted with 0.2 mL of MeOH:H_2_O (40:60, *v*/*v*), centrifuged in an Ultrafree^®^-MC centrifugal device for 5 min at 14,000× *g* at 4 °C, and transferred into vials for injection into the LC-MS system. OTA ^13^C_20_ at 5 μg/kg was used as an internal standard for OTA and AFM1. Matrix-matched calibration curves were constructed for quantification on blank samples in the range of 0.3 to 20 μg/kg for OTA and 0.04 to 2 μg/kg for AFM1.

### 4.4. Determination and Quantification: LC-MS/MS Analysis

The LC-MS/MS analysis was performed using a Waters Acquity UPLC system coupled with a Xevo^®^ TQ-XS triple quadrupole mass spectrometer equipped with an electrospray ionization (ESI) interface. Chromatographic separation was achieved using an Acquity UPLC HSS T3 column (1.8 µm × 2.1 mm × 100 mm) with a guard column of the same material. The mobile phase consisted of two mixtures: H_2_O/MeOH/acetic acid (94/5/1, *v*/*v*/*v*) (MP A) and MeOH/H_2_O/acetic acid (97/2/1, *v*/*v*/*v*) (MP B), both containing 5 mM ammonium acetate. The total analytical run time was 18 min following the gradient shown in Table 3. An injection volume of 10 μL was used. The capillary voltage was 3.2 kV, and nitrogen was used as the desolvation gas. Source and desolvation temperatures were set at 120 and 350 °C, respectively. Mass spectrometric detection was performed in positive mode, with specific transitions optimized for each mycotoxin (Table 4). Data acquisition and processing were conducted using MassLynx and TagetLynx^®^ version 4.1 software (Waters Corp., Milford, MA, USA).

### 4.5. Method Validation

The validation of the analytical method was performed in accordance with the guidelines outlined in SANTE/12089/2016 and Commission Decision (EC) No 2002/657 as guidance [19,20]. Due to the unavailability of reference materials, spiked blank samples were utilized for validation. The goodness-of-fit of the linear regression (coefficient of determination, R^2^), limit of detection (LOD), calibration range expressed as the lower limit of quantification (LLOQ) and upper limit of quantification (ULOQ), recovery (%), intra-day precision (repeatability, RSDr, CV %), and inter-day precision (intermediate precision, RSDR, CV %) were calculated based on the results of 4 days of analysis in which three calibration curves were constructed and run each day. The linearity and the goodness-of-fit of the linear regression were assessed by calculating the coefficient of determination (R^2^) [39] between the spiked concentrations of the analytes and the corresponding instrument response. Matrix effects, namely signal suppression enhancement (SSE), were assessed following the approach described in [40]. Specificity was determined by evaluating the presence of interfering chromatographic peaks in representative blank samples. The carry-over was evaluated by injecting injection solvent after the highest calibration point into the LC-MS/MS system. The method was designed and validated in the most complex matrix (firm-ripened cheese) to be broadly applicable to other cheese matrices. Moreover, when analyzing the different types of cheese, a matrix-match calibration curve was done in the same matrix that was being analyzed. Examples of spiked and naturally contaminated samples with OTA and AFM1 can be found in Supplementary Material S2.

### 4.6. Cheese Consumption, Exposure Assessment and Risk Characterization

The chronic food consumption of firm-ripened cheese for consumers only in g/kg bw/day was extracted from the Belgian national food consumption survey of 2014, available in the Comprehensive European Food Consumption Database [41]. Data reporting the consumption of firm-ripened cheese by other children (36 months-9 years), adolescents (10–17 years), and adults (18–64 years) was converted to kg/kg bw/day. The reported mean consumption values were 0.00074 kg/kg bw/day for other children and 0.00194 kg/kg bw/day for high consumers. Adolescents had a mean consumption of 0.00051 kg/kg bw/day, with high consumers at 0.00136 kg/kg bw/day. Adults exhibited a mean consumption of 0.0004 kg/kg bw/day, while high consumers had a consumption of 0.00098 kg/kg bw/day.

Contamination data were generated in this research project and incorporated into the Food Safety 4 EU risk assessment tool [42]. The lower bound (LB) and upper bound (UB) approach was employed, with the LB replacing samples below the LOQ with 0 and the UB using the LOQ. The average contamination in LB was 0.364 µg/kg, and, in UB, it was 0.648 µg/kg. The deterministic exposure assessments were conducted by multiplying the average and high consumption values by the mean concentration in LB and UB scenarios. The risk characterization was achieved through the MoE calculated as BMDL_10_/chronic exposure, based on the BMDL_10_ of 14.5 µg/kg bw/day for OTA considered for neoplastic effects and 0.4 µg/kg bw/day and a potency factor of 0.1 for AFM1 [16,26].

## Figures and Tables

**Table 1:** Results of the method validation for the quantification of ochratoxin A (OTA) and aflatoxin M1 (AFM)1in cheese. The performance of the method is reported as the determination coefficient (R^2^), limit of detection (LOD), calibration range—expressed as the lower limit of quantification (LLOQ)–upper limit of quantification (ULOQ,) interval—signal suppression enhancement (SSE, %), recovery (%), repeatability (RSD_r_, %), and intermediate precision (RSD_R_, %) at two levels.

Mycotoxin	R^2^	LOD (µg/kg)	Calibration Range (µg/kg)	SSE (%)	Recovery (%)		RSD_r_ (%)		RSD_R_ (%)	
			LLOQ–ULOQ		LLOQ	ULOQ	LLOQ	ULOQ	LLOQ	ULOQ
OTA	0.998	0.1	0.34–20.00	−88	104.9	102.1	15	3	12	7
AFM1	0.997	0.01	0.04–2.00	−66	104.6	101.8	18	8	14	11

OTA: Ochratoxin A; AFM1: aflatoxin M1.

**Table 2:** Prevalence, range, and mean concentration of OTA and AFM1 in different cheese categories.

Category	OTA	AFM1
**Spreadable cheeses**		
Prevalence (%)	0	0
Range (µg/kg)	n.d.	n.d.
Mean concentration (µg/kg)	n.d.	n.d.
**Cheese substitutes**		
Prevalence (%)	0	0
Range (µg/kg)	n.d.	n.d.
Mean concentration (µg/kg)	n.d.	n.d.
**Firm-ripened cheeses**		
Prevalence (%)	17	50
Range (µg/kg)	0.4–4.8	LOD/LOQ-0.09
Mean concentration (µg/kg)	2.2	0.1

OTA: Ochratoxin A; AFM1: aflatoxin M1; n.d.: Not detected. Mean concentration of positive samples only.

**Table 3:** Chromatographic gradient used for the analysis of cheese.

Time (min)	Mobile Phase A (%)	Mobile Phase B (%)
0.00	95	5
7.00	35	65
11.00	25	75
13.00	1	99
14.00	1	99
14.10	95	5
18.00	95	5

**Table 4:** Mass spectrometric parameters of the different analyzed mycotoxins in the XEVO-TQXS.

Mycotoxin	Retention Time (min)	Precursor Ion (m/z)	Molecular Ion	Cone Voltage (V)	Product Ions (m/z) ^b^	Collision Energy (eV)
AFM1	6.08	329.05	[M + H]^+^	40	273.1 (Q)	40
					229.1 (C)	22
OTA	9.51	404.0	[M + H]^+^	40	239.0(Q)	22
					358.0 (C)	15
OTA^13^C_20_ ^a^	9.51	424.4	[M + H]^+^	40	250.1 (Q)	25
					377.2 (C)	25

^a^ Internal standard. ^b^ Product ions: (Q) transition used for quantification, (C) transition used for confirmation. OTA: ochratoxin A; AFM1: aflatoxin M1; OTA ^13^C_20_: ^13^C labelled ochratoxin A.

## Data Availability

The original contributions presented in this study are included in the article and supplementary materials. Further inquiries can be directed to the corresponding author.

## Supplementary Materials

The following supporting information can be downloaded at: https://www.mdpi.com/article/10.3390/toxins16120547/s1; Supplementary Material S1: Ingredient list of the different cheeses; Supplementary Material S2: Figure S1: Chromatogram of a blank sample spiked with ochratoxin A at a concentration of 0.3 μg/kg; Figure S2: Chromatogram of an analyzed sample contaminated with ochratoxin A at concentration 1.4 μg/kg; Figure S3: Chromatogram of a blank sample spiked with aflatoxin M1 at a concentration of 0.04 μg/kg; Figure S4: Chromatogram of an analyzed sample contaminated with aflatoxin M1 at concentration 0.05 μg/kg.
