# Supplementary material for: Ochratoxin A and AFM1 in Cheese and Cheese Substitutes: LC-MS/MS Method Validation, Natural Occurrence, and Risk Assessment
Source: Toxins (Basel). 2024 Dec 18;16(12):547. doi: 10.3390/toxins16120547 (PMC11679095; doi:10.3390/toxins16120547)
Supplement: Supplementary file 1 [file toxins-16-00547-s001.zip › Supplementary S2.pdf]

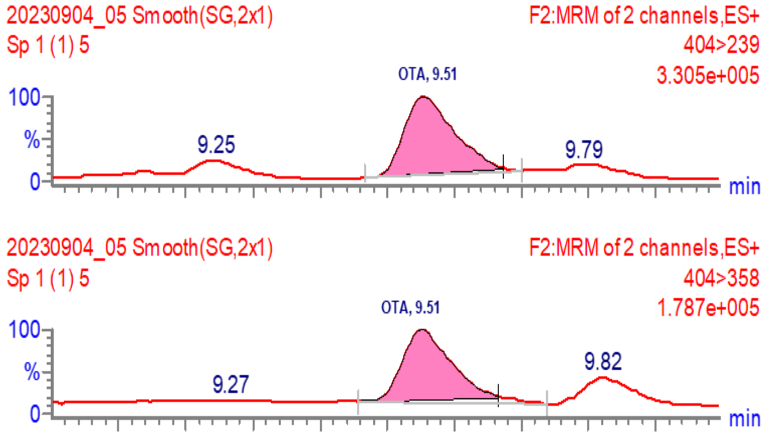

Figure S1. Chromatogram of a blank sample spiked with ochratoxin A at a concentration of 0.3 μg/kg.

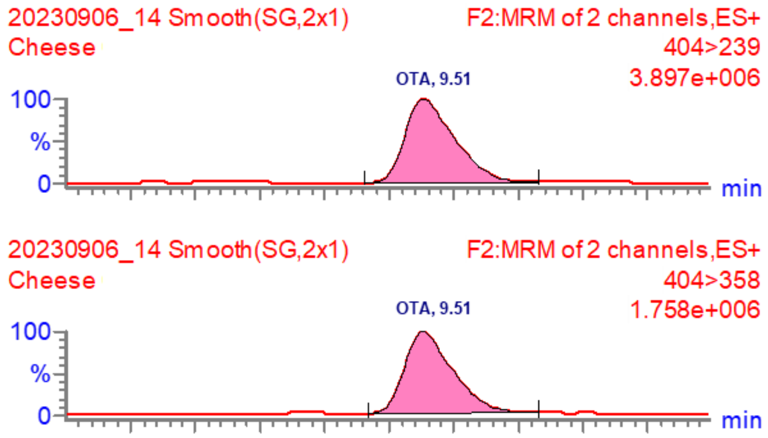

Figure S2. Chromatogram of an analyzed sample contaminated with ochratoxin A at concentration 1.4 μg/kg.

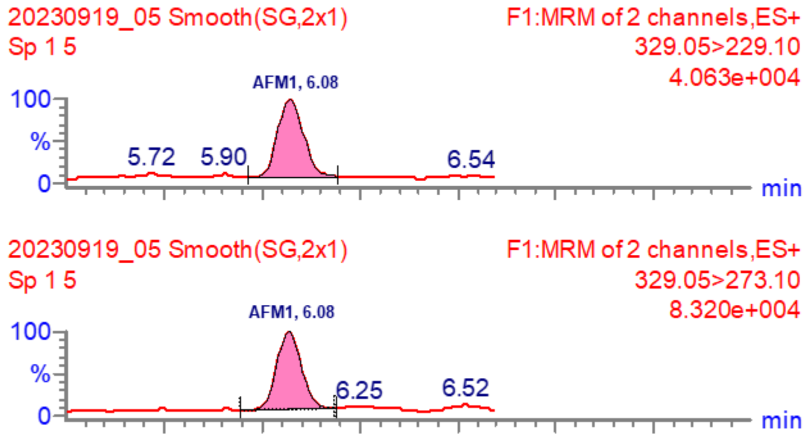

Figure S3. Chromatogram of a blank sample spiked with aflatoxin M1 at a concentration of 0.04 μg/kg.

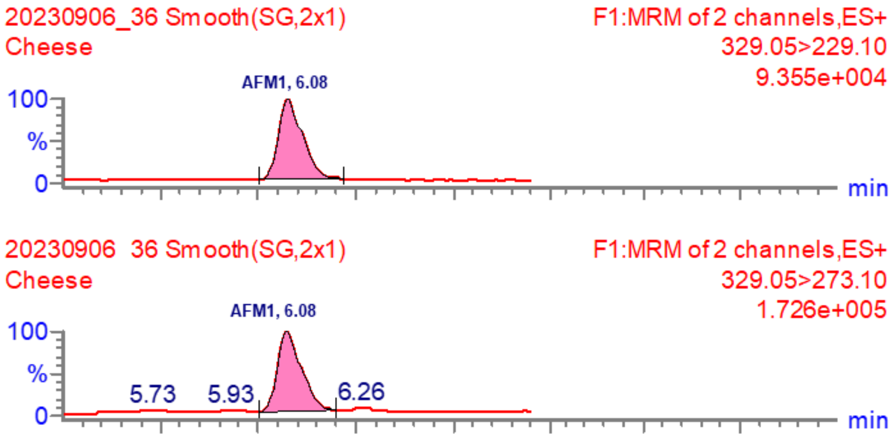

Figure S4. Chromatogram of an analyzed sample contaminated with aflatoxin M1 at concentration 0.05 μg/kg.
